# The public cancer radiology imaging collections of The Cancer Imaging Archive

**DOI:** 10.1038/sdata.2017.124

**Published:** 2017-09-19

**Authors:** Fred Prior, Kirk Smith, Ashish Sharma, Justin Kirby, Lawrence Tarbox, Ken Clark, William Bennett, Tracy Nolan, John Freymann

**Affiliations:** 1 University of Arkansas for Medical Sciences, Little Rock, Arkansas 72205, USA; 2 Emory University, Atlanta, Georgia 30322, USA; 3 Leidos Biomedical Research Inc. Frederick National Laboratory for Cancer Research, Frederick, Maryland 20892, USA; 4 Washington University School of Medicine, St Louis, Missouri 63110, USA

**Keywords:** Radiography, Cancer imaging, Diagnostic markers, Databases

## Abstract

The Cancer Imaging Archive (TCIA) is the U.S. National Cancer Institute’s repository for cancer imaging and related information. TCIA contains 30.9 million radiology images representing data collected from approximately 37,568 subjects. This data is organized into collections by tumor-type with many collections also including analytic results or clinical data. TCIA staff carefully de-identify and curate all incoming collections prior to making the information available via web browser or programmatic interfaces. Each published collection within TCIA is assigned a Digital Object Identifier that references the collection. Additionally, researchers who use TCIA data may publish the subset of information used in their analysis by requesting a TCIA generated Digital Object Identifier. This data descriptor is a review of a selected subset of existing publicly available TCIA collections. It outlines the curation and publication methods employed by TCIA and makes available 15 collections of cancer imaging data.

## Background & Summary

The Cancer Imaging Archive is a web-accessible information resource designed to promote research reproducibility and encourage reuse of data. Through rigorous data curation TCIA supports the creation of larger patient cohorts by combining sets from multiple projects and trials. TCIA encourages and supports cancer-related open science communities by hosting and managing image collections and providing searchable metadata repositories to facilitate collaborative research^1^.

The primary data managed by TCIA are radiology images of cancer, e.g., Computed Tomography (CT), Magnetic Resonance Imaging (MRI), and Positron Emission Tomography (PET) imaging studies. These may have come from clinical trials, investigator initiated research, or clinical repositories and may have been collected under a single collection protocol or multiple protocols representing current clinical practice. The image data are organized into collections by cancer type. Collections contain data submitted by one or more sites. Each collection may also include additional information depending on the type of study that originated the data. Thus, TCIA collections may include image annotations and markup (e.g., regions of interest and associated statistics), digitized pathology slides and associated pathology results, clinical data including outcomes, image feature sets generated either by expert human observers or quantitative analytic algorithms, radiation therapy structures, doses, and plans and links to external databases that contain related genomic and proteomic data.

TCIA offers a web-based query and download mechanism and an application programming interface for identifying and downloading information^2,3^. To facilitate data sharing, an increasing number of journals encourage authors to include linkages to the data that were used to create the concluded results described in a publication. As a service to the community, TCIA has the ability to create persistent Digital Object Identifiers (DOIs)^4^ to reference a set of data hosted within its archive. A TCIA data DOI provides a mechanism for researchers to cite and to directly acquire TCIA data. Data DOIs created by TCIA fall into two categories: ‘Primary Data’ which include radiology and pathology images together with supporting data (e.g. demographics, clinical outcomes, treatment information); and ‘Analysis Results’ (e.g. tumor segmentations, radiomics features, derived image maps, radiologist assessments) that are derived from TCIA Primary Data. An analysis DOI may reference a subset of a TCIA collection or superset, which spans multiple collections.

## Methods

Researchers are encouraged to submit cancer-imaging data to TCIA. A National Cancer Institute (NCI) committee reviews submission proposals and prioritizes collections based on data quality, scientific value and the potential for reuse or re-analysis. Once it is determined that a data set is of sufficient merit to include in the archive, TCIA staff initiate the submission process. An important part of the process includes working with image submitters to properly de-identify the submitted images while retaining scientifically valuable metadata^5^. The de-identification process is managed by a staff of subject matter experts and provides a uniform mechanism (software, procedures) that has been reviewed and approved by the Institutional Review Board of the TCIA hosting institution.

Following industry best practices, TCIA uses a standards-based approach to the de-identification of images stored in the Digital Imaging and Communications in Medicine (DICOM) format to insure they are free of protected health information (PHI). The TCIA de-identification process ensures that the HIPAA de-identification standard is met by following the Safe Harbor Method as defined in section 164.514(b)(2) of the HIPAA Privacy Rule (Code of Federal Regulations Title 45). DICOM Standard PS3.15 2016a—Security and System Management Profiles^6^ defines how to correctly de-identify DICOM objects. TCIA data submission software incorporates the ‘Basic Application Confidentiality Profile’ which is amended by inclusion of the following profile options: Clean Pixel Data Option, Clean Descriptors Option, Retain Longitudinal With Modified Dates Option, Retain Patient Characteristics Option, Retain Device Identity Option, and Retain Safe Private Option. At the submitting site, a script removes or modifies DICOM tags to remove known sources of PHI in compliance to the above referenced DICOM profile.

Once images are transferred to TCIA’s intake server, image curation assures image quality and data integrity. A data curator visually inspects every DICOM image. Although the DICOM standard specifies the type of values that should be stored in standard DICOM tags conformance to the standard by vendors is not always achieved. In other cases, data may have been manipulated as part of the research protocol and DICOM tags rewritten prior to being sent to TCIA. TCIA uses the Posda open source framework^7,8^ to implement its curation process. Posda is a set of curation workflow tools developed to provide a mechanism to both insure the scientific utility of data and to eliminate protected health information, while improving the scalability of curation workflow and tracking all corrections made to submissions. Posda leverages the dciodvfy (http://www.dclunie.com/dicom3tools/dciodvfy.html) validation tool as well as its own DICOM validation rule set to test DICOM files for conformance. It supports inspection and editing of PHI and allows editing of the data to correct inconsistencies found particularly in complex DICOM data objects such as those associated with Radiation Therapy. The current Posda release supports: automatic detection of subject, study, and series level DICOM inconsistencies, semi-automatic correction of inconsistencies; detection and correction tools for RTSTRUCT, RTDOSE, and RTPLAN linkage errors; checks for duplicate SOP Instance UID’s; semi-automated detection and correction of PHI; detection of duplicate pixel data for different subjects.

After Posda processing is complete, visual inspection assures that the images are free from PHI burned into the pixel data and that the image matches the body part of interest. To insure that the DICOM tags are free of PHI, unique values from all DICOM tags are extracted and written to a report. A data curator reviews the report and denotes any residual PHI that should be removed or modified prior to publishing the data. DICOM consistency checks and validation are performed to insure that the published data are useful to the scientific community. Data curation is essential to assure the ability to find and reuse data stored in an information repository^9^. The TCIA curation team verifies completeness of the received collection, full removal of all PHI, proper labeling of the information to facilitate retrieval, and proper linkages among components of the collection, e.g. images, patient demographics and outcomes, annotations, segmentation objects, radiation therapy treatment plans. The full curation process has been documented elsewhere^5,10^.

Data collections are persistently and reliably stored in a high-availability private cloud managed by the TCIA team. Each collection is documented on linked wiki pages that describe the contents of the collection, relevant publications, and information needed for proper reuse of the data. Analytic results derived from the stored images or related information, e.g. clinical trial data, demographics, associated pathology results may also be accessed from the wiki pages.

TCIA uses the DataCite system^11^ to reference data collections. DataCite leverages the Digital Object Identifier (DOI) infrastructure, which is widely used in citing scholarly articles. TCIA provides publicly available DOIs for each collection, and will also provide DOIs for subsets of data stored within TCIA on request. All information related to a TCIA DOI must be persistently managed by TCIA. Each TCIA Data DOI links to a landing page that provides a summary of the data, a detailed description as provided by the submitter, citation information, relevant metadata, a mechanism to download the latest version of the data, and a mechanism to download previous versions of the data.

**Figure 1:**
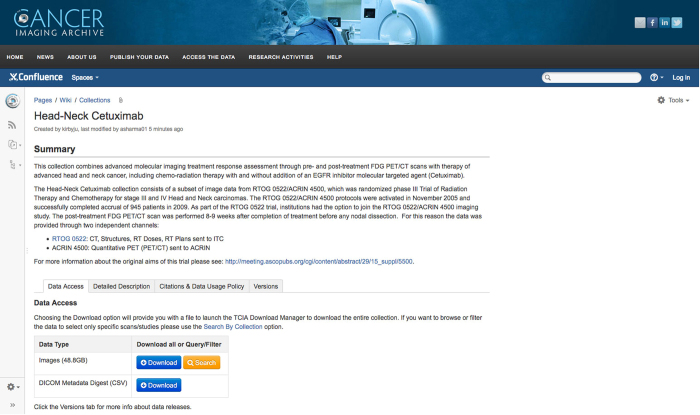
The Head-Neck Cetuximab landing page is an example of the standard landing page format referenced by each data citation. Data may be accessed by clicking the Download button. Image metadata may also be accessed using the associated Download button for DICOM Metadata Digest (CSV).

Figure 1 illustrates the standard format for TCIA DOI landing pages. Image data can be downloaded by clicking on the Download link. The download mechanism will create a two level folder structure on the receiving computer in which the first level represents the DICOM study and the second level is the DICOM series. This is a very commonly known DICOM hierarchy and is meant to be consumed by DICOM workstations and applications. Optionally, metadata for each image in the hierarchy can be downloaded as a comma separated value file using the DICOM Metadata Digest (CSV) link.

Unless explicitly noted all collections are freely available to browse, download, and use for commercial, scientific and educational purposes as outlined in the Creative Commons Attribution 3.0 Unported (CC BY 3.0) License.

## Data Records

Head-Neck Cetuximab Collection [Data Citation 1] combines pre- and post-treatment FDG PET/CT scans of subjects undergoing therapy for advanced head and neck cancer, including chemo-radiation therapy with and without addition of an EGFR inhibitor targeted agent (Cetuximab). This collection consists of data for a subset of the subjects enrolled in a Radiation Therapy Oncology Group (RTOG), American College of Radiology Imaging Group (ACRIN) clinical trial, RTOG 0522/ACRIN 4500, which was a randomized phase III Trial of Radiation Therapy and Chemotherapy for Stage III and IV head and neck carcinomas^12^. PET/CT image data for 111 subjects are included as well as Radiation Therapy (RT) Structure Sets, Dose and Therapy plans are included for 101 of the subjects.

CT Colonography Collection [Data Citation 2] contains prone and supine DICOM CT images of the colon from 825 subjects collected under ACRIN PROTOCOL 6664 (ref. 13), ClinicalTrials.gov Identifier: NCT00084929 (refs 14,15). The collection contains a portion of the data from that trial that has been publicly released as well as spreadsheets identifying positive and negative polyp cases (polyp descriptions and locations).

LIDC-IDRI Collection [Data Citation 3] contains diagnostic and lung cancer screening thoracic CT data collected and analyzed by The Lung Image Database Consortium (LIDC) and image database resource initiative^16^. CT image cases representing 1010 subjects are associated with expert annotations and image markup stored as XML objects that can be optionally downloaded with the images^17^. Care was taken to acquire the data under a standard protocol and nodule annotations were created by consensus among multiple expert observers. When using this data in scientific publications or technical reports please cite^16^ and add the following attribution: ‘The authors acknowledge the National Cancer Institute and the Foundation for the National Institutes of Health, and their critical role in the creation of the free publicly available LIDC/IDRI Database used in this study.’

Lung Phantom Collection [Data Citation 4] contains CT images of the FDA anthropomorphic thorax phantom^18^ with 12 phantom lesions of different sizes, shapes, and densities scanned on a 64-detector row scanner (LightSpeed VCT, GE Healthcare, Milwaukee, WI). The CT scanning parameters were 120 kVp, 100 mAs, 64×0.625 collimation, and pitch of 1.375. The images were reconstructed with the lung kernel using 1.25 mm slice thickness. This data may not be used for commercial purposes.

Phantom FDA Collection [Data Citation 5] contains CT images of the FDA anthropomorphic thorax phantom^18^ configured with 8 nodule layouts scanned on a Philips Mx8000 IDT (Philips Healthcare, Andover, MA) and a Siemens Somatom Definition 64 scanner (Siemens Medical Solutions USA, Inc., Malvern, PA). Each nodule layout was scanned using 16 acquisition protocols that varied combinations of effective dose, pitch, and slice collimation, and were reconstructed with varying combinations of slice thicknesses and reconstruction kernels^19^. Ten exposures were acquired for each imaging protocol on each scanner. The data should not be used for commercial purposes. When using this data in scientific publications or technical reports please cite^18^.

LungCT-Diagnosis Collection [Data Citation 6] contains diagnostic contrast enhanced chest CT scans for 61 subjects collected at diagnosis and prior to surgery. The data were retrospectively acquired from clinical image repositories. The objective of the study was to extract prognostic image features that characterize lung adenocarcinomas^20^. Associated excel format files contain data to identify representative tumor images, pathology staging information and survival data.

NaF PROSTATE Collection [Data Citation 7] contains F-18 labeled Sodium Fluoride (NaF) PET/CT images of 9 patients with prostate cancer and suspected or known bone involvement. Some of the patients had 2 baseline studies within 14 days of each other (with no interventions). Many have follow-up PET/CT imaging performed following therapy (varied) at 6+/−2 months and 12+/−2 months. Data acquisition parameters are described in ref. 21.

QIN PET Phantom Collection [Data Citation 8] contains PET phantom scans originally utilized by the Quantitative Imaging Network PET Segmentation Challenge^2^ to assess the variability of segmentations and subsequently derived quantitative analysis results. Because these are images of a NEMA IEC Body Phantom Set Model PET/IEC-BODY/P, ground truth is known. The phantom was scanned at 2 sites using different imaging systems (Siemens, GE), with four image sets per site.

The RIDER Collections represent a targeted data collection used to generate an initial consensus on how to harmonize data collection and analysis for quantitative imaging methods funded by The National Cancer Institute. The Reference Image Database to Evaluate Therapy Response (RIDER)^22^ data were transferred to TCIA upon completion of the project. Data collection methods are described in the Combined RIDER White Paper Report^23^. The available collections are referenced by the following RIDER data citations.

RIDER Phantom PET-CT Collection [Data Citation 9] contains repeat measurements of a Sanders Medical (Knoxville, TN) PET phantom^24^ collected to assess the uniformity of clinical imaging instrumentation at various sites. A complete description of the phantom and scanning protocols is included in the Combined RIDER White Paper Report^23^. The collection includes 20 PET-CT data sets.

RIDER Phantom MRI Collection [Data Citation 10] contains repeat measurements of a phantom to measure sources of variance in DCE MRI^25^. The phantom used for all data acquisitions was a version of the EuroSpin II Test Object 5 as distributed by Diagnostic Sonar, Ltd (Livingston, West Lothian, Scotland). A complete description of the phantom and scanning protocols is included in the cited material. Four MRI systems were used for data acquisition and a total of 10 data sets were collected^23,26^.

RIDER Neuro MRI Collection [Data Citation 11] contains repeated MRI scans of 19 subjects diagnosed with recurrent glioblastoma. Repeat measurements were made 1 or 2 days apart using the same imaging system. All 19 subjects had Dynamic Contract Enhanced (DCE) scans on the same 1.5 T MRI. The citation includes details of the acquisition protocol and additional details are included in the Combined RIDER White Paper Report^23^. Diffusion tensor imaging (DTI) data were obtained from seventeen of the 19 subjects with whole brain coverage, 12 directions, at a b value of 1000 smm^−2^. Contrast-enhanced 3D FLASH data was collected in the sagittal plane on all 19 subjects and contrast-enhanced 3D FLAIR was collected on the 17 subjects who also had repeat DTI measurements. Before transmission to TCIA, all image sets with 1 mm isotropic voxel size were ‘defaced’ using MIPAV software or manually.

RIDER Lung PET-CT Collection [Data Citation 12] represents a longitudinal study of 244 subjects. The data were collected to enable the quantification of sources of variance in PET/CT imaging with the goal of defining the minimum effect size that should be used in the design of clinical trials using PET measurements as end points^23^.

RIDER Lung CT Collection [Data Citation 13] contains images from repeated Lung CT scans (‘coffee break experiment’) for 32 subjects diagnosed with non-small cell lung cancer. The collection also contains a spreadsheet of identified lesions^27,28^. Each subject underwent 2 CT studies on the same scanner using the same imaging protocol. The scans were completed within 15 min of one another.

RIDER Breast MRI Collection [Data Citation 14] contains coffee break repeat DCE MRI data sets on 5 subjects diagnosed with primary breast cancer. Subjects received 2 MRI scans within a 15 min interval http://www.med.umich.edu/cmi/pdf/ISMRM2009Poster.pdf (2009). All data were collected prior to the onset of neoadjuvant chemotherapy.

ISPY1 Collection [Data Citation 15] contains MRI data from 222 Breast cancer patients enrolled in the ACRIN 6657 trial and its companion study Cancer and Leukemia Group B (CALGB) study 150007 (ref. 29). ACRIN 6657/CALGB 150007 were prospective studies to predict response to neoadjuvant therapy. Collectively, CALGB 150007 and ACRIN 6657 formed the basis of the multicenter **I**nvestigation of **S**erial Studies to **P**redict **Y**our **T**herapeutic **R**esponse with **I**maging and mo**L**ecular **A**nalysis (I-SPY TRIAL) breast cancer trial. The data in this collection represents the complete MRI dataset from I-SPY 1/ACRIN 6657 plus clinical and outcomes data in spreadsheet format.

Table 1 provides a summary of key attributes of the collection cited.

## Technical Validation

The TCIA curation process assures completeness of the acquired data and optimizes this data for discovery and reuse, but it does not validate the collection process or the analyses performed and published by the research community. In cases where the data managed by TCIA were collected as part of a clinical trial or controlled experiment, additional acquisition protocol consistency may be enforced. Other collections reflect the variance of acquisition protocol representative of standard clinical practice. Feedback generated by reuse of data by researchers provides information that may be used by TCIA to perform additional quality review. Such feedback or requests for additional scientific information on a collection may be addressed to help@cancerimagingarchive.net.

## Additional information

**How to cite this article**: Prior, F. *et al.* The public cancer radiology imaging collections of The Cancer Imaging Archive. *Sci. Data* 4:170124 doi: 10.1038/sdata.2017.124 (2017).

**Publisher**’**s note**: Springer Nature remains neutral with regard to jurisdictional claims in published maps and institutional affiliations.

## Supplementary Material



## Figures and Tables

**Table 1:** Summary of Cited Data Collections.

**Collection**	**Data Size (GB)**	**Modalities**	**Anatomic Site**	**Protocol Refs.**
Head-Neck Cetuximab	48.8	PT (positron emission tomography), CT (computed tomography), RTSTRUCT (Radiation Therapy Structure Set), RTDOSE (Radiation Therapy Dose), RTPLAN (Radiation Therapy Plan)	Head, Neck	^30^
CT Colonography	462.6	CT (computed tomography)	Colon, Abdomen	^13^
LIDC-IDRI	124	CT (computed tomography) DX (digital radiography) CR (computed radiography)	Lung, Chest	^16^
Lung Phantom	127.5	CT (computed tomography)	Lung	See Data Record
Phantom FDA	125.4	CT (computed tomography)	Lung	^19^
LungCT-Diagnosis	2.5	CT (computed tomography)	Lung	Std. of Care
NaF PROSTATE	12.9	PT (positron emission tomography), CT (computed tomography)	Prostate	^21^
QIN PET Phantom	0.25	PT (positron emission tomography)	Body Phantom	^31^
RIDER Phantom PET-CT	0.69	PT (positron emission tomography), CT (computed tomography)	Chest Phantom	^23^
RIDER Phantom MRI	3.4	MR (magnetic resonance Imaging)	Chest Phantom	^23,26^
RIDER Neuro MRI	7.3	MR (magnetic resonance Imaging)	Brain	^23^
RIDER Lung PET-CT	83.27	PT (positron emission tomography), CT (computed tomography)	Lung	^23^
RIDER Lung CT	7.55	CT (computed tomography)	Lung	^23^
RIDER Breast MRI	0.402	MR (magnetic resonance Imaging)	Breast	^23^
ISPY1	76.2	MR (magnetic resonance Imaging), SEG (Segmentation)	Breast	^29,32^
